# A comparative pilot study on Gram-negative bacteria contaminating the hands of children living in urban and rural areas of Indonesia versus Germany – A suitable monitoring strategy for diarrhea risk assessment?

**DOI:** 10.3389/fmicb.2023.1152411

**Published:** 2023-04-03

**Authors:** Debi Frina Simanjuntak, R. Lia Kusumawati, Oliver Bader, Carsten G. K. Lüder, Ortrud Zimmermann, Uwe Groß

**Affiliations:** ^1^Institute for Medical Microbiology and Virology, University Medical Center Göttingen, Göttingen, Germany; ^2^Department of Microbiology, Faculty of Medicine, Universitas Sumatera Utara, Medan, Indonesia

**Keywords:** Gram-negative bacteria, Enterobacterales, hand hygiene, children, diarrhea, Indonesia

## Abstract

Diarrhea is the second leading cause of death mainly effecting young children. Often it is the result of fecal-oral pathogen transmission. We aimed to investigate whether monitoring the prevalence of Gram-negative bacteria on the hands of asymptomatic children is suitable as an indicator of fecal contamination of the environment in their playground. We compared the prevalence of Gram-negative bacteria on the hands of children, who live in the German city of Göttingen, an urban area in a high-income country, with the situation in Medan as an urban area and Siberut as a rural area both in the middle-income country Indonesia. A total of 511 children at the age of 3 months to 14 years were asked to put their thumb print on MacConkey agar, which was used to screen for the presence of Gram-negative bacteria. These were subsequently identified by using MALD-TOF mass spectrometry and classified into the order Enterobacterales, Pseudomonadales, and others. The highest burden of hand contamination was found in children from rural Siberut (66.7%) followed by children from urban Medan (53.9%), and from urban Göttingen (40.6%). In all three study sites, hand contamination was lower in the youngest (<1 year) and oldest age groups (10–14 years) and highest in the age group 5–9 years. Bacteria of the order Enterobacterales possibly indicating fecal contamination were most prevalent in Siberut (85.1%) followed by Medan (62.9%) and Göttingen (21.5%). Most facultative and obligate gastrointestinal pathogens such as *Escherichia coli* (*n* = 2) and *Providencia rettgeri* (*n* = 7), both being members of the order Enterobacterales, as well as *Aeromonas caviae* (*n* = 5), and *Vibrio cholerae* (*n* = 1) both belonging to other orders were nearly exclusively identified on the hands of children in Siberut. This result was not surprising, because hygienic conditions were lowest in Siberut. Only one isolate of *A. caviae* was found in Medan, and no facultative gastrointestinal pathogen was identified on the hands of children from Göttingen. Our pilot study therefore indicates that investigating hands of children for the prevalence of Gram-negative bacteria using selective media are a helpful method to monitor hygienic conditions, and thereby assess the risk for diarrhea-causing bacterial pathogens in the environment.

## Introduction

The World Health Organization (WHO) noted that diarrhea is the second leading cause of death, affecting more than 800,000 people each year (Liu et al., 2012). Although diarrhea can have many causes, the most common causes are food poisoning or, more generally, ingestion of bacterial gastrointestinal pathogens that contaminate food or drinking water. Human or animal feces or waste containing such pathogens might contaminate water. Polluted water used for soil irrigation and fisheries also can contain gastrointestinal bacterial pathogens (Prüss-Ustün et al., 2017). In addition, several viruses and parasites, food intolerances or intoxications as well as vegetative symptoms such as anxiety, drugs, irritability triggered by antibiotics or laxatives are among factors that may lead to diarrhea.

The fecal-oral route for which fecal contamination of hands often is an important pre-requisite mostly transmits pathogens causing diarrhea. A recent review reported that hands are more likely contaminated in low/lower-middle-income countries (Cantrell et al., 2023). Fecal indicator bacteria concentrations have been shown to be associated with adverse health outcomes in young children (Goddard et al., 2020). Indeed, children under the age of five are prone to diarrhea, especially in low-income countries (Cantrell et al., 2023). It is therefore particularly important for this age group to have a clean home and environment. Child feces management is complex but may have an impact on the levels of fecal bacteria in children’s household environments (Bauza et al., 2020). Good hygiene practices outside the home, such as at work, following hygiene-promoting behavior, such as washing hands regularly with soap and clean water, and drinking clean, filtered water to prevent the spread of fecal pathogens are important rules for all households (Badowski et al., 2011).

The vast majority of bacteria transiently colonizing human skin are Gram-positive and – with the exception of enterotoxin-producing *Staphylococcus aureus* or *Bacillus cereus* – they rarely cause diarrhea (Lambers et al., 2006). In contrast, several Gram-negative bacterial species are well-known gastrointestinal pathogens, such as members of the order Enterobacterales (e.g., *Salmonella* spp., *Escherichia coli*, *Shigella* spp., and *Yersinia* spp.) and other Gram-negative bacteria, such as, e.g., *Vibrio cholerae* or *Aeromonas caviae*. Infection with these bacteria often results in diarrhea. In addition to diarrhea, a negative correlation between *E. coli* in play spaces and on hands of children with development outcome scores has been demonstrated (George et al., 2022). As expected, higher levels of hand contamination with *E. coli* were found among mobile and less among very young and immobile children (Parvez et al., 2019). Likewise, toddlers who are more active and crawl on the floor a lot show a more exploratory behavior, so they end up putting most of the things they find in their mouths, such as soil or even feces (Ngure et al., 2013).

Since children are very often affected by diarrhea, we aimed to investigate the prevalence of Gram-negative bacteria on the hands of children in different geographic regions to prove the following two main hypotheses. First, Gram-negative bacteria from fecal contaminations of the environment are more common in rural areas with low hygiene conditions than in urban areas with conditions of better hygiene (Shakoor et al., 2012; Cantrell et al., 2023). Second, the prevalence of these bacteria on the hands of children differs based on age-dependent activities, such as play and sport, to help draw conclusions about the risks of developing diarrhea in these children.

In order to investigate the relationship between different environments and different educational and hygienic standards on bacterial contamination of hands, we investigated the situation in the German city of Göttingen and two Indonesian regions, the city of Medan and the island Siberut. Göttingen and Medan represent urban areas, while Siberut is a rural area. On one hand, we selected Göttingen and Medan as urban areas because of their higher population density, middle/upper class social structure and little agricultural activity. On the other hand, Siberut as an isolated island whose population subsists on agricultural activity and whose infrastructure is not so well developed represents all the characteristics of a rural area. In addition, child feces management practices may be different between the study sites.

## Materials and methods

### Study design and patients

The Ethical Committees of the University of North Sumatra (No. 229/KOMET/FK USU/2011), the Health Department of North Sumatra (No. 440.800/2040IX/2011), and the University Medical Center Göttingen (No. 29/3/11) approved this prospective, descriptive group-related multi-center study which differentiated according to gender and age. Sampling at the three study sites was performed from April to October 2011 from children who were met either at home, in the kindergarten, or at school, respectively. Since the first author was born in Indonesia, she easily explained the aim and design of the study to the parents and their children. The parents were of sound mind to give informed consent in accordance with the Declaration of Helsinki. The children up to 14 years of age were assigned to the following groups: I (children under 1 year), II (between 1 and 4 years), III (between 5 and 9 years), and IV (between 10 and 14 years). This classification was based on the assumption that children under the age of 1 are still under the control of their mothers/parents, while between the ages of 1 and 4 years a period begins when children begin to walk and explore their surroundings more closely. Children between the ages of 5 and 9 are then in a phase where they are increasingly mastering formal thought processes, and finally children between the ages of 10 and 14 begin to experience health and environmental socialization with increasing awareness. In this study, we have postulated that the bacterial flora on the children’s hands differs based on age-dependent activities and allows the assessment of risks for causing diarrheal diseases in children. Since no comparable studies have been described before this study has been performed, and since this study was intended as a pilot study, we were unable to execute a valid sample size calculation. Instead, we decided to investigate at least 500 children. However, we judge this sample size as adequate for investigating hand contamination of children, because similar studies that were performed later included sample sizes of 169–468 children (Otsuka et al., 2019; Pasaribu et al., 2019; Vishwanath et al., 2019).

### Situation analysis of the three study sites

Göttingen is well known for its old university (founded in 1734) and is located approximately in the middle of Germany. In 2011, the census recorded 116,052 residents with a density of 992 people per km^2^ (source: State Office for Statistics Lower Saxony). The mild climate with yearly mean temperature of 8.4°C is continental with moderate humidity and yearly mean precipitation of 628 mm. The German health system, various forms of support for families with children, unemployment benefits and the pension system guarantee a high standard of living and health. The income of most people is based on their employment. Access to and usage of sanitation facilities such as toilets for child feces management are common and defecation in open places is a very rare event. Sufficient sewage treatment plants as well as controlled clean water are present (Table 1). In most cases, mothers care for their children under the age of one. Childminders or similar facilities often paid by the parents eventually supervise children up to 3 years. With the age of three, children usually attend state-subsidized day care centers. At the age of six, they enter free primary education. For leisure, the children spend their time playing games out- and inside and rest at home. The elementary school consists of four school years. Depending on their performance, students can subsequently choose between three different forms of schooling guaranteed by the governments of the federal states for up to nine additional years.

**TABLE 1:** Characteristics of typical households in the study areas.

	Göttingen	Medan	Siberut
Household crowding	1–2 persons/room	2–3 persons/room	3–8 persons/room
Predominant toilet type	Flush toilet in private room	Squat toilet in private room	Open toilet for >1 family
Source of drinking water	Tap water (regularly monitored)	treated tap water	Sand/stone-filtered water in containers
Hand washing stations	Yes	Yes	No

We selected two sites in Indonesia to capture both a densely populated urban area and an underdeveloped rural area in a middle-income country. Medan is the capital of North Sumatra province on the Indonesian island of Sumatra and covers 2,651 km^2^ with a population of 2,117,224 people (2011 census) at a population density of 7,986 per km^2^ (source: Badan Pusat Statistik Provinsi Sumatera Utara). Its tropical rainforest climate without a significant dry period has a yearly mean precipitation of 2,263 mm and an average temperature of 27°C. The city of Medan has a variety of neighborhoods that are dominated by different ethnic groups, such as by Deli, Malay, Batak, Javanese, Minang, Acehnese, Chinese, Indians, and other immigrant ethnic groups. There is a popular state university (University of Sumatera Utara, founded in 1956). Medan is also known for its culinary tradition. Several large rivers flow through the city and flow into the Straits of Malacca; one of which is the river Deli. Nearly 40 municipal health centers and 41 sub-centers (3–4 so-called puskesmas per district) support the healthcare system. These puskesmas are still the most common frequented health centers of the city. More than 1,405 additional smaller centers are responsible for basic medical services such as immunization, prevention/preventive measures against malnutrition in children, pregnancy assistance, family planning, and a variety of measures for the education and information of the population in the area of health. Medan also has a so-called type B hospital with free referral-based medical services specifically for the poorer population (the Pirngadi Hospital). Childcare takes place exclusively in the families: mothers and other family members nearly always take care for babies under the age of 1 year. Subsequently, most 4–6 years-olds visit facilities for early childhood education and development (ECD). From age 7–14, the children should then complete their primary and secondary education. Most children have access to toilets or use these regularly (Table 1).

**FIGURE 1:**
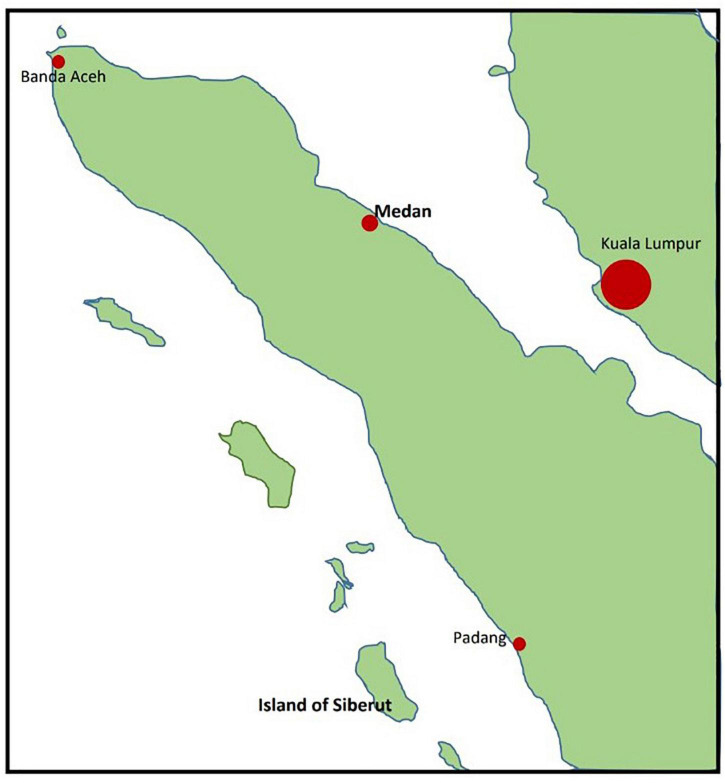
Overview of the location of the two Indonesian study sites.

**FIGURE 2:**
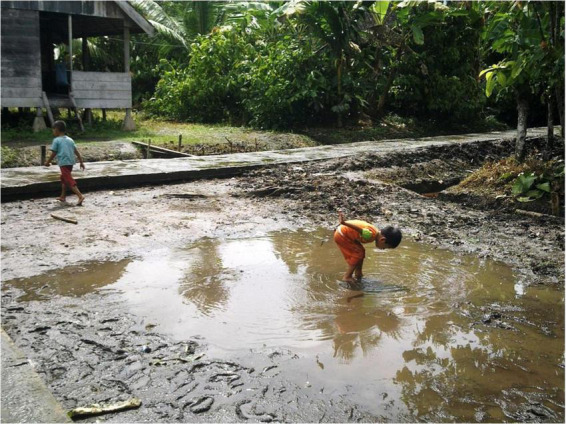
Barefoot children play in the mud. A typical longhouse of Policoman (island of Siberut) is seen in the background.

The second Indonesian study site was the village of Policoman in the north of Siberut Island. This island belongs to the island world of Mentawai and is located in the Indian Ocean West of the Indonesian island of Sumatra. An overview map of the Indonesian study sites is shown in Figure 1. The total area of Mentawai covers 6,011 km^2^ with a population of 77,078 (2011 census) and a population density of 13 people per km^2^ (source: Badan Pusat Statistik Kabupaten Kepulauan Mentawai). The capital is Tua Pejat (in Sipora District). Siberut Island has a tropical rainforest climate with an average annual temperature of 29°C and 5,950 mm of precipitation. It has been declared as biosphere reserve by the UNESCO since 1981. As a region without industrialization, there is no modern infrastructure, factories or other industrial activities, and people live there in a simple, nature-centered way of life. Most people belong to the Sakuddei tribe and live in so-called *uma* (longhouses). In the center of the island, people grow rice for their personal use. Goods needed for consumption are bought once a week from Padang, the capital of West Sumatra. Mentawai has one hospital and seven community health centers. One of the centers with inpatient treatment options is located in Muara Sikabaluan and is in charge of the 7,774 residents of the North of Siberut island (source: Pusat Data dan Informasi Kesehatan Kabupaten Kepulauan Mentawai Provinsi Sumatera Barat). The village of Policoman in the north of Siberut Island consists of 106 families with typically two to six children per family. The government takes care of the health education of the local population through monthly activities. The island of Siberut can only be reached from the city of Padang by ferries that run once a week with an approximate driving time of 10 h. From the ferry terminal, it takes another two and a half hours by speedboat to reach Policoman. The diet and lifestyle of the population there is generally on established agriculture. Usually coconut and cocoa trees are planted on commercial fields far from the farmers’ homes that can only be reached by boat, which takes about half an hour. On the other hand, the fields that are used for their own needs are closer to the settlement and produce cassava (manioc) and bananas. Pig farming is another source of income for residents to purchase the goods and services (primarily schooling) they need on a daily basis. Additional sources of nutrition are protein-yielding fishing and keeping chickens, which are found in almost all families. In addition, dried cocoa beans for sale in Padang are another source of income for the villagers. A constant supply of electricity is not guaranteed. Therefore, about five families always share one private power generator. For cooking, they collect firewood from the area around the farm. Fresh water is hard to come by because the village lies along the swampy coast. The groundwater is not clear, but rather brownish, reddish or even black with a slightly sour taste. For cooking, water is drawn from wells, which is filtered using smaller boulders and sand. Unfiltered water from the wells is also used for personal hygiene (bathing and showering) as well as for washing dishes and laundry. Although toilets exist that, however, are used by more than one family, open defecation or throwing child feces into an open field is common (Table 1). The village of Policoman has a kindergarten for young children and an elementary school. After school, children usually play outdoors (Figure 2). The younger children aged around four to six usually follow the older ones. Children over the age of seven engage in local games such as tag, climb coconut trees, archery, and frolic in the swampy coastal area, often swimming in the river. After lunch and school, the older children (over 12 years old) help their parents with the work in the fields. In the kindergarten, most of the children do not wear shoes. Parents’ knowledge of sanitary necessities is still low. In elementary schools, wearing shoes was ordered by the government. There are three shops in the area that sell groceries (including packaged groceries). On school days, during breaks, some small retailers offer snacks and drinks on or in front of the school premises. What exactly is offered there is not controlled by local authorities. After school, the students usually romp around outside and practice climbing and canoeing, for example.

### Laboratory methods

The aim and procedures of the study were explained to the parents and children. Upon parental consent, each child slightly pressed a thumb on a MacConkey agar plate (bioMérieux, Nürtingen, Germany) without having washed the hand before. The agar plates were then immediately sealed and stored in a portable cooler. Subsequently, the samples were taken to the local laboratory to be incubated for 48 h at 37°C. Up to five microbial colonies growing on MacConkey agar were recultured on Columbia blood agar (bioMérieux, Nürtingen, Germany) for additional 24 h at 37°C for increasing bacterial biomass under high-nutrient conditions. Using a swab, the recultured colonies were then placed in an agar gel transport system (Oxoid, Basingstoke, UK) for later identification. Time for transportation was not critical because we increased bacterial biomass before and focused on Gram-negative environmentally stable bacteria. In addition, we had tested long-time viability of putative Gram-negative contaminants before starting this study. Subsequently, the bacteria were identified in the laboratories of the Institute of Medical Microbiology and Virology in Göttingen/Germany using MALDI-TOF mass spectrometry (MALDI Biotyper database V10.0, Bruker Daltonics, Bremen, Germany) as described (Noll et al., 2020).

### Statistical analysis

Data were entered in two-way frequency tables and visualized by frequency interaction plots using Statistica version 13.5 (TIBCO Software Inc., Palo Alto, USA). Relationship between variables were analyzed by univariate Pearson Chi-square test. *p*-Values of less than 0.05 were considered statistically significant. To account for multiple significance tests on the same data set, *p*-values were Bonferroni-adjusted (*p*_adj._).

## Results

In this prospective pilot study, Gram-negative bacteria contaminating the hands of 511 children were determined. From Göttingen, 160 children were examined, 180 children from Medan, and 171 children from the Island of Siberut, subdivided according to age and sex as shown in Table 2.

**TABLE 2:** Number of participating children (M, male; F, female).

Group (age in years)	Germany	Indonesia	
	**Göttingen**	**Medan**	**Siberut**
I (<1)	12 (6 M, 6 F)	20 (10 M, 10 F)	14 (8 M, 6 F)
II (1–4)	77 (40 M, 37 F)	80 (31 M, 49 F)	77 (29 M, 48 F)
III (5–9)	40 (17 M, 23 F)	40 (18 M, 22 F)	40 (23 M, 17 F)
IV (10–14)	31 (7 M, 24 F)	40 (14 M, 26 F)	40 (20 M, 20 F)
Total number	160 (70 M, 90 F)	180 (73 M, 107 F)	171 (80 M, 91 F)

### Bacterial classification

Gram-negative bacteria found on the hands of children were classified into the order Enterobacterales, Pseudomonadales, and other Gram-negative bacteria. The order Enterobacterales consisted mainly of bacteria belonging to the families Enterobacteriaceae (*n* = 104), Morganellaceae (*n* = 35), Yersiniaceae (*n* = 18), and Erwiniaceae (*n* = 15), whereas the order Pseudomonadales were mainly composed of bacteria belonging to the families Pseudomonadaceae (*n* = 174) and Moraxellaceae (*n* = 55). The group of other Gram-negative bacteria was very heterogeneous and consisted mainly of *Stenotrophomonas* spp. (*n* = 11), *Comamonas* spp. (*n* = 9), *Aeromonas* spp. (*n* = 6), and several other species including *Vibrio cholerae*.

### Prevalence of Gram-negative bacteria that contaminate the hands of children

**FIGURE 3:**
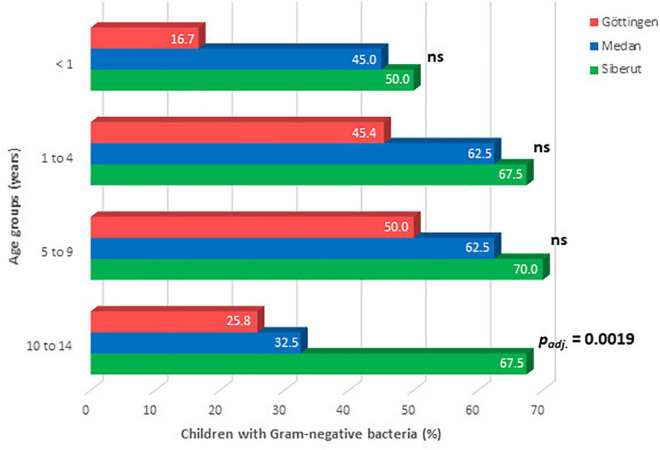
Age-related contamination of children’s hands with Gram-negative bacteria in the three study regions. Data were analyzed by Chi-square test, with Bonferroni-adjustment of *p*-values (ns, not significant).

Overall, significant differences for hand contamination were identified between the three study sites and between the age groups (Table 3). No differences in hand contaminations were identified between female and male children. The highest burden of hand contamination with Gram-negative bacteria was found in children from rural Siberut followed by children from urban Medan and then from Göttingen. Chi-square tests indicated statistically different hand contaminations among children from Göttingen and Siberut (*p*_adj._ < 0.0001), Göttingen and Medan (*p*_adj._ = 0.0436), and also among children from Medan and Siberut (*p*_adj._ = 0.0436). Thus, hand contamination with Gram-negative bacteria differed between children from rural Siberut and both urban study sites, reflecting the low standard of hygiene in rural Siberut. However, it also suggested differences in the hygienic conditions between the urban study sites in Indonesia and Germany. In all three study sites, hand contamination was lower in the youngest (<1 year) and highest in the age group 5–9 years with significant differences between the study sites in the oldest age group 10–14 years (Figure 3). In Göttingen, Gram-negative bacteria were found on the hands of 65 children out of 160 examined (40.6%; Table 3). Most affected children were in the age group 5–9 years of age (Figure 3). In comparison, in the urban Indonesian region of Medan, Gram-negative bacterial isolates were identified on the hands of 97 out of 180 examined children (53.9%; Table 3). Like in Göttingen, most affected children were in the age group 5–9 years, but also in the age group 1–4 years (Figure 3). In contrast, in the rural region of Siberut, Gram-negative bacteria were found on the hands of 114 out of 171 children (66.7%). With 70.0%, the highest burden of hand contamination was also identified in 5–9 years old children from Siberut (Figure 3).

**TABLE 3:** Association between hand contamination and sociodemographic factors.

Variables	Gram-negative bacteria		*p*-value
	**Contaminated *n* (%)**	**Non-contaminated *n* (%)**	
Study site			<0.0001
Göttingen, Germany	65 (40.6)	95 (59.4)	
Medan, Indonesia	97 (53.9)	83 (46.1)	
Siberut, Indonesia	114 (66.7)	57 (33.3)	
Age (years)			0.0037
<1	18 (39.1)	28 (60.9)	
1–4	137 (58.5)	97 (41.5)	
5–9	73 (60.8)	47 (39.2)	
10–14	48 (43.2)	63 (56.8)	
Sex			0.9210
Female	155 (53.8)	133 (46.2)	
Male	121 (54.3)	102 (45.7)	

We also compared hand contamination with certain age groups across the different study sites. Gram-negative bacteria were found on the hands of 16.7% of the children under the age of 1 year in Göttingen (Figure 3 and Supplementary Table 1). In contrast, the hands of nearly every second infant from Medan (45%) and from Siberut (50%) showed contamination with Gram-negative bacteria. This indicates that the child-related hygiene at this age is still too low in both Indonesian study sites. In children aged 1–9 years, there was no major difference between Göttingen, Medan, and Siberut with regard to the detection of Gram-negative bacteria (Figure 3). In contrast, when looking to schoolchildren between the ages of 10 and 14 years, Gram-negative bacteria were found in the minority of children in Göttingen (25.8%) as well as in Medan (32.5%), but in the majority of children from Siberut (67.5%; *p*_adj._ = 0.0019; Figure 3).

### Classification of bacteria

Gram-negative bacteria were found on the hands of 65 children (40.6%) from Göttingen (Table 3). Bacteria of the order Pseudomonadales predominated with 57 isolates (87.7% of hand-contaminated children identified in Göttingen; Table 4) consisting of 13 different *Pseudomonas* species, three different *Acinetobacter* species, and two different *Moraxella* species. Most prevalent was *Pseudomonas putida*. The order Enterobacterales followed with 14 isolates (21.5% of hand-contaminated children from Göttingen; Table 4) consisting of *Enterobacter cloacae* complex, *Pantoea agglomerans* and two different *Serratia* species. In fact, with 10 isolates, *P. agglomerans* was most prevalent in Göttingen. Other bacteria (*n* = 10, 15.4%; Table 4) were composed of a mixture of seven different bacterial species that usually are not associated with gastrointestinal infections.

**TABLE 4:** Association between hand-contaminating bacterial isolates and sociodemographic factors.

Variables	Gram-negative isolates *n* (%)^1^					
	**Enterobacterales**		**Pseudomonadales**		**Others**	
	**Yes**	**No**	**Yes**	**No**	**Yes**	**No**
**Study site**						
Göttingen, Germany	14 (21.5)	51 (78.5)	57 (87.7)	8 (12.3)	10 (15.4)	55 (84.6)
Medan, Indonesia	61 (62.9)	36 (37.1)	81 (83.5)	16 (16.)	12 (12.4)	85 (87.6)
Siberut, Indonesia	97 (85.1)	17 (14.9)	91 (79.8)	23 (20.2)	45 (39.5)	69 (60.5)
	*p* < 0.0001		*p* = 0.3978		*p* < 0.0001	
**Age (years)**						
<1	13 (72.2)	5 (27.8)	10 (55.6)	8 (44.4)	1 (5.6)	17 (94.4)
1–4	96 (70.1)	41 (29.9)	117 (85.4)	20 (14.6)	32 (23.4)	105 (76.6)
5–9	37 (50.7)	36 (49.3)	63 (86.3)	10 (13.7)	12 (16.4)	61 (83.6)
10–14	26 (54.2)	22 (45.8)	39 (81.3)	9 (18.7)	22 (45.8)	26 (54.2)
	*p* = 0.0201		*p* = 0.0127		*p* = 0.0004	

^1^Data indicate numbers of bacterial isolates and percentages of hand-contaminated children of the respective study group. Data were analyzed by Chi-square test. *P*-values of less than 0.05 were considered statistically significant.

In Medan, Gram-negative bacteria were identified on the hands of 97 children (53.9%; Table 3). Like in Göttingen, most prevalent was the order Pseudomonadales with 81 isolates (83.5% of hand-contaminated children from Medan; Table 4) and four different *Acinetobacter* species. From the three different *Pseudomonas* species identified, *Pseudomonas stutzeri* was most prevalent with 64 isolates. In contrast to Göttingen, much more bacteria from the order Enterobacterales were present on the hands of children from Medan (61 isolates, 62.9%; Table 4); most prevalent were bacteria belonging to the *E. cloacae* complex (*n* = 33). The other bacteria (*n* = 12, 12.4%) consisted of six different species including one case with *Aeromonas caviae*.

The situation was significantly different in the rural study site, the Island of Siberut. Here, two third of the examined children (*n* = 114, 66.7%; Table 3) presented with hand contaminations caused by Gram-negative bacteria. In contrast to the two urban study sites, it was the order Enterobacterales that was predominately found on the hands of children from Siberut (*n* = 97, 85.1% of all hand-contaminated children identified in Siberut; Table 4). However, like in Medan, with 37 isolates, *E. cloacae* complex was most dominant. We also identified seven isolates of *Providencia rettgeri*, four of them in the age group 10–14 years. In addition, *E. coli* could be found on the hands of two children, each in the age group 5–9 years. Since further typing was not performed, it remains unclear whether these bacteria belonged to a specific pathovar (e.g., EHEC, EPEC, or ETEC). Furthermore, 91 isolates (79.8% of hand-contaminated children from Siberut) of the order Pseudomonadales and seven different *Acinetobacter* spp. were found. Like in Medan, *P. stutzeri* was most prevalent with 43 isolates. In contrast to the two urban study sites, with 45 isolates (39.5%; Table 4), much more other Gram-negative bacteria were contaminating the hands of children, who live on the island of Siberut. Among those bacteria, *Myroides odoratimimus* (*n* = 9), *Stenotrophomonas* spp. (*n* = 7), and *A. caviae* (*n* = 5) were most prevalent.

For the prevalence of Enterobacterales, significant differences were calculated between Siberut and Göttingen (*p*_adj._ < 0.0001), between Medan and Göttingen (*p*_adj._ < 0.0001) but also between the two Indonesian study sites Siberut and Medan (*p*_adj._ = 0.0006). Most prevalent within the order Enterobacterales was *E. cloacae* complex in Siberut (15.9%) as well as in Medan (21.4%). In contrast, in Göttingen the most prevalent species within this order was *P. agglomerans* (12.3%). With 45 isolates from 52 hand-contaminated children (86.5%) and 6 out of 7 positive children (85.7%), most contaminations with Enterobacterales have been found in 1–4 years old children from Siberut and in children under the age of 1 from Siberut, respectively (Supplementary Table 1). No significant differences between the three study sites were found for Pseudomonadales (Table 4, *p* = 0.3978), whereas other Gram-negative bacteria were significantly more often identified on the hands of children from Siberut in comparison to those from Göttingen (*p*_adj._ = 0.0023) but also in comparison to the children from Medan (*p*_adj._ < 0.0001). For Pseudomonadales and other Gram-negative bacteria, the highest burden of contamination were present in the age groups of 5–9 and 10–14 years old children from Siberut, respectively (Supplementary Table 1). Nevertheless, three 1-year-old children from Siberut presented themselves with *A. caviae*. This facultative gastrointestinal pathogen was also identified in a 3-years-old and a 6-years-old child from Siberut and in one child from the age group 1–4 years from Medan, respectively. In addition, other putative diarrhea-causing bacteria were also identified on the hands of children in Siberut; with seven isolates *P. rettgeri* was most prevalent. In addition, two isolates of *E. coli*, and one isolate of *V. cholerae* were found on the hands of 5-years-old children from Siberut. Since this was a pilot study, no further subtyping of bacterial isolates had been carried out.

In Siberut, 57.9% of bacteria-positive children had an average of two different Gram-negative bacterial species on their hands. In contrast, both the rate of affected children and the mean number of different bacterial species were lower in Medan and in Göttingen (*p* < 0.0001; Table 5). Furthermore, mixed bacterial populations also differed between different age groups (*p* = 0.0144; Table 5) with highest detection rates in children between 1 and 4 years.

**TABLE 5:** Prevalence of hand-contaminating mixed bacterial populations and their association with sociodemographic factors.

Variables	Positive children *n*	Bacterial isolates *n*	Mixed population^1^ *n* (%)		Bacterial isolates/children^2^
			**Yes**	**No**	
**Study site**					
Göttingen, Germany	65	81	15 (23.1)	50 (76.9)	1.25
Medan, Indonesia	97	154	44 (45.4)	53 (54.6)	1.59
Siberut, Indonesia	114	233	66 (57.9)	48 (42.1)	2.04
			*p* < 0.0001		
**Age (years)**					
<1	18	24	5 (27.8)	13 (72.2)	1.33
1–4	137	245	75 (54.7)	62 (45.3)	1.79
5–9	73	112	26 (35.6)	47 (64.4)	1.53
10–14	48	87	19 (39.6)	29 (60.4)	1.81
			*p* = 0.0144		

^1^Children with mixed bacterial population. Numbers in parenthesis indicate percentages of positive children within the respective study group.

^2^Data indicate average number of isolates per positive children. Data were analyzed by Chi-square test. *P*-values of less than 0.05 were considered statistically significant.

## Discussion

This study aimed in proving the hypotheses that Gram-negative bacteria from fecal contaminations of the environment are more common on the hands of children who live in rural areas with low hygiene conditions than in urban areas with conditions of better hygiene and that the prevalence of these bacteria on their hands differs based on age-dependent activities. For this, we investigated the hands of 511 children aged up to 14 years who either live in the German city of Göttingen, the Indonesian city of Medan, and the rural Indonesian island of Siberut for the presence of Gram-negative bacteria. Indeed, we found the highest burden of hand contamination in children from rural Siberut followed by children from urban Medan and from urban Göttingen. In all three study sites, hand contamination was lower in the youngest (<1 year) and oldest age groups (10–14 years) and highest in the age group 5–9 years. Enterobacterales possibly indicating fecal contamination were most prevalent in Siberut followed by Medan and Göttingen. As expected, we identified most facultative and obligate gastrointestinal pathogens nearly exclusively on the hands of children in Siberut as hygienic conditions were lower there than in the urban study areas.

We were interested to understand, whether unwashed hands of children might serve as vehicles for transmitting pathogens from the environment, especially under low hygienic conditions and different practices of child feces management (Bauza et al., 2020, 2023). When analyzing hands of children in an urban slum of Indonesia, Otsuka et al. (2019) noticed fecal contamination in most children. However, that study focused only on *E. coli* as an indicator for fecal contamination. In contrast, we included all Gram-negative bacteria as representatives for gastrointestinal pathogens and investigated their prevalence on the hands of children who live in one of three study areas that represent different living and hygiene conditions in Germany and Indonesia in order to identify potential risks for development of diarrhea. Apart from the four most common pathogens (rotavirus, *Cryptosporidium*, ETEC, and *Shigella*) *Aeromonas*, *V. cholerae* O1, and *Campylobacter jejuni* were important pathogens causing moderate to severe diarrhea in children (9,439 diarrhea sufferers and 13,129 controls) younger than 5 years of age in several countries in sub-Saharan Africa and South Asia (Kotloff et al., 2013).

Whereas Gram-negative bacteria were present in less than half of the children from Göttingen, every second child from Medan and even two out of three children from Siberut presented themselves with those bacteria. This main result suggests that children may be exposed to higher levels of fecal contamination in the environment in the two Indonesian study sites compared to the German site. The frequency of mixed contamination can also be an indicator of poor hygiene especially in Siberut. Indeed, both the highest rate of affected children and the highest average number of different Gram-negative bacterial species were found in Siberut.

Overall, the data compiled in this study suggest that knowledge, attitudes, and practices around hygiene behavior are one of many factors influencing children’s exposure to fecal bacteria (Mourad et al., 2019). Baker et al. (2011) confirm that the level of education and the degree of hygiene determine the degree of general health in the respective population. In addition, access to und usage of sanitation facilities, such as toilets are important pre-requisites for preventing diarrhea (Bauza et al., 2023). In Göttingen, as in Medan, the percentage of children in the age group 10–14 years with no evidence of Gram-negative bacterial hand contamination was higher than in the age groups 1–4 and 5–9 years. This result may indicate that children in these two urban cities have also been educated on basic hygiene measures and behaviors at school over time and that the children apparently behave quite well when it comes to hand hygiene. The most plausible reason for this seems to be the fact that they attend school and, as pre-adolescents, have already acquired a good understanding of cleanliness and hygiene. In contrast, the situation in Siberut turned out to be significantly worse: the percentage of children of the examined age groups without evidence of a Gram-negative bacterial contamination was only about 30% in the groups of 1–14 years old. This result was most likely due to the living conditions in Siberut; there is a lack of clean (let alone running) water and flush toilets.

The development of cognitive, emotional, and social skills and abilities of children depends on the psychosocial development they experience in their respective socio-cultural environment. Toddlers who are more active and crawl on the floor a lot show a more exploratory behavior, so they end up putting most of the things they find in their mouths. If the environmental conditions are of low hygiene standard developing diarrhea is therefore a constant threat. When they grow up and play outside their home, they have correspondingly more movement possibilities. During this period, especially the children’s imaginations develop in the form of games and they try out many things with increasing independence. Their curiosity about things in their immediate environment grows without them being aware of potential dangers. When children reach preschool age, they usually learn increasingly more (pre-) school activities. They cope with tasks and play in groups with peers. At this stage, children learn basic morals and the gradual control of their own impulsiveness. At about 6 years, the mindset or way of thinking of children is still holistic and quite easy. With progressive development, however, up to the 10th year of life, they learn to think more and more in a logical-analytical way (Mills, 2013).

The abstract and formal way of thinking increases with the age of puberty and becomes accompanied by emotional adjustments and alignments as well as the formation of introspective skills. Pellegrini (1992), who investigated differences in childhood activities outside the home, concluded that pre-adolescent children of younger ages generally prefer to play away from home longer than older pre-adolescent ones. Boys between the ages of 3 and 4 years prefer climbing frames and swings, while girls are much more likely to play in the sandbox (Holmes and Procaccino, 2009). When they start visiting school, a short period of socialization begins of students within their peer group without close parental supervision, and this has influence on their social, emotional and cognitive development (Kirylo et al., 2010). Blatchford et al. (2003) observed that children 7–8 years old use their break times very often for social interactions. Blatchford (1996) describes the changes in patterns in children’s play behavior during the transition from primary school to secondary school. At 11 years of age, interactive games such as soccer dominate the play behavior of boys. A significant change in the behavior of children occurs between 11 and 16 years: children who like to actively participate in games such as football, hunting, and catching in elementary school increasingly start to initiate conversation and contact-seeking and -keeping behavior with friends during and until the end the secondary levels.

The highest number of different Gram-negative bacterial isolates on the hands of children in all study sites (in Siberut also in 10–14-years olds) was found in the age groups 1–4 and 5–9. This result confirms those of Mills (2013), according to which 1–10-years-old children are in a behavioral phase of a more exploratory character: they constantly want to explore and try new things themselves and are very keen on discoveries. This behavior corresponds to their growing abilities of imagination and imagination in play as well as in the development of increasing autonomy and self-control in their motor skills, but also in feeding and excretion.

The large number of different Gram-negative bacteria on the children’s hands made it useful to classify them according to orders: 1. Enterobacterales, 2. Pseudomonadales, and 3. other Gram-negative bacteria. Within this classification, we wanted to determine whether environmentally stable diarrheal pathogens, especially *E. coli* and other obligate gastrointestinal pathogens such as *Salmonella*, *Shigella*, *Yersinia*, or *V. cholerae* would be more frequently detectable in tropical regions (Medan and Siberut) than in the non-tropical region (Göttingen).

Four *Comamonas aquatica* isolates and four *Comamonas testosteroni* isolates were found on the hands of children in Siberut, but only one *C. testosteroni* isolate in Medan. Comamonas lives in both aquatic environments, such as sewage sludge, and terrestrial (Liu et al., 2017). Myroides was not reported in either Göttingen or Medan, but nine Myroides isolates were found on hands of children in Siberut. These bacteria can also be found in water-rich areas, e.g., in freshwater fish (Maull et al., 2013). The finding of many species of Comamonas and Myroides in Siberut is apparently due to the behavior of the children: they usually play barefoot on the ground or in the mud and swim and play in the river, catch fish in the sea, paddle a canoe to save things to transport to the settlements, and the like.

Similar to our study, Pasaribu et al. (2019) investigated 468 school children between 6 and 12 years of age in a rural village of North Sumatra for soil-transmitted helminth infection (STH). In that study and similar to the situation in Siberut, playing with soil increased the risk, whereas hand washing habits and latrine usage decreased the risk of STH infection.

Potential diarrhea-causing bacterial pathogens have only been detected in Indonesia. The detection of *E. coli* in water samples is generally an indicator for fecal contamination. In fact, *E. coli* was only found on the hands of children in Siberut. Aeromonas are Gram-negative bacteria commonly found in water-rich tropical and subtropical areas (particularly in waters with dead fishes). Through contamination of the water, these bacteria can infect both animal and human intestines, but can also multiply extra-intestinally (Janda and Abbott, 2010; Pessoa et al., 2022). One isolate of *A. caviae*, which can cause watery diarrhea (Senderovich et al., 2012), was found on the hand of a child from Medan, while five of these isolates were found on hands of children in Siberut. In addition, one isolate of *V. cholerae* was found on the hand of a child in Siberut, although there was no outbreak of diarrhea in Siberut during the time of this investigation.

Vishwanath et al. (2019) used blood and MacConkey agar to examine the hands of 200 school children aged 7–15 in the rural area of Kelambakkam, India. More than 95% of the children had commensal bacteria such as coagulase-negative staphylococci (CNS) and aerobic spore-formers. Other bacteria isolated were *Acinetobacter* spp. (36.5%), *Pseudomonas* spp. (4%), *Klebsiella* spp. (3.5%), *Enterococcus* spp. (2%), *E. coli* (2%), *Flavobacterium* spp. (1.7%), and *Enterobacter* spp. (0.75%). Commensal bacteria such as aerobic spore formers and CNS were more frequent in female children (*p* = 0.32), while *Acinetobacter*, *Pseudomonas*, *E. coli*, and *Flavobacterium* were found comparatively more frequently in male children (*p* < 0.05). The most prevalent bacteria detected in India are similar to those of our study, however, the prevalence rates of *Pseudomonas* are higher in our study sites. *Pseudomonas* was mainly detected in Göttingen (45.6%) and Medan (47.4%) and less frequently in Siberut (27.5%). These bacteria can occur as opportunistic pathogens typically causing nosocomial infections. Obligate gastrointestinal pathogens such as *Salmonella*, *Shigella*, *Yersinia*, or *Campylobacter* were neither found in the study of Vishwanath et al. (2019) nor in our study. However, the comparison of the studies indicates that worse hand hygiene is practiced in the Indonesian study sites compared to the Indian study site.

It is estimated that approximately 20% or 51 million people of the Indonesian population defecate in open areas such as fields, bushes, and beaches (Hirai et al., 2016). The government of Indonesia promoted handwashing with soap through its national health care program with the goal of ending the practice of defecation in open spaces in 20,000 villages by 2019 (Hirai et al., 2016). The five pillars of this project include eliminating the practice of open defecation, increasing the practice of washing hands with soap, improving household water supplies, and improving dirty water and garbage management. The main benefits of handwashing with soap are the reduction of diarrhea (Bartram and Cairncross, 2010).

In November 2012, UNICEF and the Indonesian government launched a 4-years project to promote sanitation and hygiene conditions in the eastern provinces of Indonesia, to expand and strengthen the already initiated national sanitation and hygiene program (Hirai et al., 2016). The result of the WASH intervention (hand washing program) in 450 schools was that pupils who were taught hygiene knowledge and practices by their teachers defecated significantly less often in the open air, shared often their knowledge with their parents and were more willing to wash their hands (Karon et al., 2017).

Knee et al. (2018) examined stool samples of 759 children aged 1–48 months in Maputo, Mozambique, independent of diarrhea symptoms, for the detection of 15 common gastrointestinal pathogens using multiplex RT-PCR. Most children (86%) had ≥1 enteric pathogen in their stool sample. The prevalence of enteric infection was positively associated with age, ranging from 71% in children 1–11 months of age to 96% in children between 24 and 48 months. The authors found a high prevalence of enteric infections, especially in children without diarrhea, and weak associations between bacterial infections and environmental risk factors, including WASH (hand washing program) interventions. Certain hygienically positive latrine conditions, including drop hole covers and sturdy latrine walls, and the presence of a faucet on site were associated with a lower risk of bacterial infections. However, only few WASH implementation studies or behavioral change interventions seemed to be established in Indonesia, as has been shown in a very recent systematic review (Satriani et al., 2022). Most of these studies were performed in the Central and Eastern provinces of Indonesia, but neither in Medan nor Siberut (Satriani et al., 2022). As a result, open defecation and the lack of regularly hand-washing procedures are still present especially in Siberut.

It is therefore not surprising that the sanitary situation in Siberut is similar to that in Maputo as not every household could afford a clean toilet and access to clean water at the time. A 10th of the houses in Policoman village on the island of Siberut had to share the wells. Indeed, in agreement with our study was the result of the investigation performed by Knee et al. (2018) that children <1 year had the lowest percentage of Gram-negative bacteria compared to the children of the other age groups. Likewise, the prevalence of the age-dependent detection of Gram-negative bacteria in Göttingen, Medan, and Siberut was predominantly positively associated with age.

Shah et al. (2017) analyzed stool samples from 1,060 children in a suburban and a rural study site in western Kenya. Diarrhea-causing *E. coli* strains were detected most frequently (32.8/44.1%). We were also able to demonstrate a higher prevalence of Enterobacterales in Indonesia, especially in the rural region of Siberut (85.1% of hand-contaminated children), but less often in urban Göttingen (21.5%, Siberut versus Göttingen *p*_adj._ < 0.0001). This finding could be explained by the fact that the standard hygiene in Siberut is still relatively low compared to Göttingen. Potential bacterial diarrhea-causing pathogens in Kenya were also identified in children examined in Siberut, namely *P. rettgeri* (*n* = 7), *A. caviae* (*n* = 5), *E. coli* (*n* = 2), and *V. cholerae* (*n* = 1). In contrast, only one isolate of *A. caviae* was found in urban Medan. In the Kenyian study, a higher hygiene discrepancy was described between the two study sites. The hygiene situation in the rural study site was similar to that of Siberut, which could be the most plausible reason for the similar bacterial findings in both locations. Over two-thirds of households in the suburban Kenyian study site had access to improved water sources, and 80% of households were connected to a sewer, septic tank or cesspool. In the rural Kenyian study site, only 27.8% of households had improved water supply and only 41.5% were connected to a sewer, septic tank, or cesspool. Therefore, open defecation in the rural study site was very common.

When analyzing 437 stool samples from children with diarrhea under the age of 5 in the capital of Sudan, Khartoum, using culture and PCR, most prevalent was *E. coli* (48%), followed by rotavirus (22%), *Giardia intestinalis* (11%), *Entamoeba histolytica* (5%), *Salmonella* (4%), *Shigella*, and *Campylobacter* (each 2%) (Saeed et al., 2015). The majority of positive samples (84%) was found in children over 2 years of age, particularly in the 4–5 years age group. The prevalence of diarrhea in children over 2 years of age was significantly higher (*p* < 0.010). In this study, EAEC was the most commonly detected type of *E. coli* in children and was present in 43% of the cases, suggesting that it is the main cause of diarrhea in Khartoum. Based on the design of our study, *Campylobacter*, viruses, and protozoan parasites were not included in monitoring hand contamination. Future studies should therefore include also PCR-based methods to detect a wider array of pathogens.

Iturriza-Gómara et al. (2019) had collected stool samples from 684 children with diarrhea aged <5 years and 527 age-matched asymptomatic controls in Malawi and tested them for 29 pathogens using PCR. At least three pathogens were detected in 71% of the cases and in 48% of the controls. The most prevalent bacterial pathogen associated with diarrhea was *E. coli* composed of the pathovars ETEC (21.2 and 8.5%), EPEC (18.0 and 8.3%), and Shigella/EIEC (in 15.8 and 5.7%). *Aeromonas* spp. could be detected in 3.9 and 1.9%, respectively. In contrast, *V. cholerae* (1.3 and 0%), *Salmonella* Typhi (1.2 and 0.4%), and *Salmonella* Typhimurium (2.3 and 0.4%) were only rarely detected. This is in contrast to our study, where *A. caviae* was more prevalent than *E. coli* in Siberut.

Differences in incidence rates of intestinal infection or diarrhea in children seem to support the results of our study whereby the lowest prevalence of hand contamination was found in the German study site, followed by the Indonesian city of Medan, and finally by Siberut with the highest prevalence rate (Figure 3). Indeed, the incidence of diarrhea in children <15 years for the study year was lowest in Germany (0.5% of all inhouse patients, Destatis, Germany) and higher in Medan (1.40%, Pirngadi Hospital, Medan). Unfortunately, no such data were available for Siberut, but there the age-dependent prevalence rates of hand contamination were in line with the observation that the highest incidence rate of diarrhea was observed in patients >5 years of age (49.0%, Sikabaluan Health Center). Routine bacteriological diagnosis revealed that *C. jejuni* is the most prevalent bacterial pathogen causing intestinal infection in Germany, whereas detailed data on intestinal pathogens in Indonesia are very limited due to the lack of sufficient laboratory diagnosis. Our test design might have missed to detect *C. jejuni*. However, this pathogen is mostly transmitted by improper kitchen hygiene during food processing and was therefore not in focus of our study.

Washing hands is an efficient way of protection against diarrhea (Ejemot-Nwadiaro et al., 2015). According to Bartram and Cairncross (2010), people with no or difficult access to clean water and poor general sanitary and hygienic conditions in resource-constrained regions of tropical and subtropical countries are at high risk of developing diarrhea or other digestive disorders. This has also recently been confirmed in a study that was performed in an Indonesian village in Timor (Agustina et al., 2021). In fact, especially in Siberut with its poor, difficult or non-existent access to clean water and low sanitary and hygienic situation, several diarrhea-causing pathogens could be identified on the hands of children, such as *E. coli*, *A. caviae*, *P. rettgeri*, and *V. cholerae*. The fact that bacteria of the order Enterobacterales were significantly more often found there in comparison to Göttingen and also in comparison to Medan, indicate that they might serve as a good indicator of the general hygienic status within a study area.

The strengths of our pilot study were the selection of a broad range of Gram-negative bacteria, rather than focusing on *E. coli* as the only fecal indicator bacterium and a comparison of pathogen distribution between different countries. A limitation of our study is its descriptive, comparative evaluation of three different contexts. Without enrollment into the comparison groups (“hygienic urban,” “unhygienic urban,” versus “unhygienic rural”) based on individual- or neighborhood-level information related to hygiene practices or environmental contamination levels, these findings cannot easily be generalized to other contexts. Another limitation is the lack of adjustment for confounding, such as socioeconomic status or investigation of specific risk factors for hand contamination. However, we aimed this study as a pilot survey in which we compared the levels of hand contamination between the different study sites. A future and extended study needs to be able to draw interferences about a causal link between specific hygienic conditions or practices and child hand contamination.

## Conclusion

Taken together, investigating hands of children for the prevalence of Gram-negative bacteria using selective media might be a helpful method to roughly monitor (i) hygienic conditions, and (ii) the risk for diarrhea-causing bacterial pathogens in the environment. Future studies should include the investigation of environmental samples and should be carried out especially in Siberut Island because there the living and hygiene conditions have not substantially been improved since this study has been performed.

## Data availability statement

The original contributions presented in this study are included in the article/Supplementary material, further inquiries can be directed to the corresponding author.

## Ethics statement

The studies involving human participants were reviewed and approved by the Ethical Committees of the University of North Sumatra (No. 229/KOMET/FK USU/2011), the Health Department of North Sumatra (No. 440.800/2040IX/2011), and the University Medical Center Göttingen (No. 29/3/11). Written informed consent to participate in this study was provided by the participants’ legal guardian/next of kin.

## Author contributions

UG had the initial idea. DFS collected the samples and performed the microbiological analyses with the support of RLK, OB, and OZ. CGKL analyzed the data statistically. UG wrote the manuscript with contributions of all other authors. All authors read and approved the final version.

## References

[B1] Agustina A. Dukabain O. M. Singga S. Wanti W. Suluh D. G. Mado F. G. (2021). Home sanitation facilities and prevalence of diarrhea for children in Oelnasi Village, Kupang Tengah sub-district. *Gac. Sanit.* 35 S393–S395. 10.1016/j.gaceta.2021.10.059 34929859

[B2] Badowski N. Castro C. M. Montgomery M. Pickering A. J. Mamuya S. Davis J. (2011). Understanding household behavioral risk factors for diarrheal disease in Dar es Salaam: A photovoice community assessment. *J. Environ. Public Health* 2011:130467. 10.1155/2011/130467 21969836 PMC3182559

[B3] Baker D. P. Leon J. Smith Greenaway E. G. Collins J. Movit M. (2011). The education effect on population health: A reassessment. *Popul. Dev. Rev.* 37 307–332. 10.1111/j.1728-4457.2011.00412.x 21984851 PMC3188849

[B4] Bartram J. Cairncross S. (2010). Hygiene, sanitation, and water: Forgotten foundations of health. *PLoS Med.* 7:e1000367. 10.1371/journal.pmed.1000365 21085694 PMC2976722

[B5] Bauza V. Majorin F. Routray P. Sclar G. D. Caruso B. A. Clasen T. (2020). Child feces management practices and fecal contamination: A cross-sectional study in rural Odisha, India. *Sci. Total Environ.* 709:136169. 10.1016/j.scitotenv.2019.136169 31905545 PMC7031693

[B6] Bauza V. Ye W. Liao J. Majorin F. Clasen T. (2023). Interventions to improve sanitation for preventing diarrhoea. *Cochrane Database Syst. Rev.* 1:CD013328. 10.1002/14651858.CD013328.pub2 36697370 PMC9969045

[B7] Blatchford P. (1996). We did more then”: Changes in pupils’ perceptions of breaktime (Recess) from 7 to 16 Years. *J. Res. Childh. Educ.* 11 14–24. 10.1080/02568549609594692

[B8] Blatchford P. Baines E. Pellegrini A. (2003). The social context of school playground games: Sex and ethnic differences, and changes over time after entry to junior school. *Br. J. Dev. Psychol.* 21 481–505. 10.1348/026151003322535183

[B9] Cantrell M. E. Sylvestre E. Wharton H. C. Scheidegger R. Curchod L. Gute D. M. (2023). Hands are frequently contaminated with fecal bacteria and enteric pathogens globally: A systematic review and meta-analysis. *ACS Environ. Au* 10.1021/acsenvironau.2c00039

[B10] Ejemot-Nwadiaro R. I. Ehiri J. E. Arikpo D. Meremikwu M. M. Critchley J. A. (2015). Hand washing promotion for preventing diarrhoea. *Cochrane Database Syst. Rev.* 9:CD004265. 10.1002/14651858.CD004265.pub4 26346329 PMC4563982

[B11] George C. M. Birindwa A. Beck S. Julian T. Kuhl J. Williams C. (2022). Fecal contamination in child play spaces and on child hands are associated with subsequent adverse child developmental outcomes in rural Democratic Republic of the Congo: REDUCE prospective cohort study. *Am. J. Trop. Med. Hyg.* 106 1141–1148. 10.4269/ajtmh.21-0706 35189587 PMC8991330

[B12] Goddard F. G. B. Pickering A. J. Ercumen A. Brown J. Chang H. H. Clasen T. (2020). Faecal contamination of the environment and child health: A systematic review and individual participant data meta-analysis. *Lancet Planet. Health* 4 e405–e415. 10.1016/S2542-5196(20)30195-9 32918886 PMC7653404

[B13] Hirai M. Graham J. P. Mattson K. D. Kelsey A. Mukherji S. Cronin A. A. (2016). Exploring determinants of handwashing with soap in Indonesia: A quantitative analysis. *Int. J. Environ. Res. Public Health* 13:868. 10.3390/ijerph13090868 27598178 PMC5036701

[B14] Holmes R. M. Procaccino J. K. (2009). Preschool children’s outdoor play area preferences. *Early Childh. Dev. Care* 179 1103–1112. 10.1080/03004430701770694

[B15] Iturriza-Gómara M. Jere K. C. Hungerford D. Bar-Zeev N. Shioda K. Kanjerwa O. (2019). Etiology of diarrhea among hospitalized children in Blantyre, Malawi, following rotavirus vaccine introduction: A case-control study. *J. Infect. Dis.* 220 213–218. 10.1093/infdis/jiz084 30816414 PMC6581894

[B16] Janda J. M. Abbott S. L. (2010). The genus *Aeromonas*: Taxonomy, pathogenicity, and infection. *Clin. Microbiol. Rev.* 23 35–73. 10.1128/CMR.00039-09 20065325 PMC2806660

[B17] Karon A. J. Cronin A. A. Cronk R. Hendrawan R. (2017). Improving water, sanitation, and hygiene in schools in Indonesia: A cross-sectional assessment on sustaining infrastructural and behavioral interventions. *Int. J. Hyg. Environ. Health* 220 539–550. 10.1016/j.ijheh.2017.02.001 28238610

[B18] Kirylo J. D. Thirumurthy V. Patte M. M. (2010). Issue in education: Can you imagine a world without recess? *Child Educ.* 87 62–63. 10.1080/00094056.2010.10521440

[B19] Knee J. Sumner T. Adriano Z. Berendes D. de Bruijn E. Schmidt W. P. (2018). Risk factors for childhood enteric infection in urban Maputo, Mozambique: A cross-sectional study. *PLoS Negl. Trop. Dis.* 12:e0006956. 10.1371/journal.pntd.0006956 30419034 PMC6258421

[B20] Kotloff K. L. Nataro J. P. Blackwelder W. C. Nasrin D. Farag T. H. Panchalingam S. (2013). Burden and aetiology of diarrhoeal disease in infants and young children in developing countries (the Global Enteric Multicenter Study, GEMS): A prospective, case- control study. *Lancet* 382 209–222. 10.1016/S0140-6736(13)60844-2 23680352

[B21] Lambers H. Piessens S. Bloem A. Pronk H. Finkel P. (2006). Natural skin surface pH is on average below 5, which is beneficial for its resident flora. *Int. J. Cosmet. Sci.* 28 359–370. 10.1111/j.1467-2494.2006.00344.x 18489300

[B22] Liu L. Johnson H. L. Cousens S. Perin J. Scott S. Lawn J. E. (2012). Global, regional, and national causes of child mortality: An updated systematic analysis for 2010 with time trends since 2000. *Lancet* 379 2151–2161. 10.1016/S0140-6736(12)60560-1 22579125

[B23] Liu T. Mao Y. Shi Y. Quan X. (2017). Start-up and bacterial community compositions of partial nitrification in moving bed biofilm reactor. *Appl. Microbiol. Biotechnol.* 101 2563–2574. 10.1007/s00253-016-8003-9 27900438

[B24] Maull K. D. Hickey M. E. Lee J. L. (2013). The study and identification of bacterial spoilage species isolated from catfish during refrigerated storage. *J. Food Process. Technol.* S11, S11–S13. 10.4172/2157-7110.S11-003

[B25] Mills C. M. (2013). Knowing when to doubt: Developing a critical stance when learning from others. *Dev. Psychol.* 49 404–418. 10.1037/a0029500 22889395 PMC3810952

[B26] Mourad K. A. Habumugisha V. Sule B. F. (2019). Assessing students’ knowledge on WASH-related diseases. *Int. J. Environ. Res. Public Health* 16:2052. 10.3390/ijerph16112052 31185642 PMC6604011

[B27] Ngure F. M. Humphrey J. H. Mbuya M. N. Majo F. Mutasa K. Govha M. (2013). Formative research on hygiene behaviors and geophagy among infants and young children and implications of exposure to fecal bacteria. *Am. J. Trop. Med. Hyg.* 89 709–716. 10.4269/ajtmh 24002485 PMC3795101

[B28] Noll C. Nasruddin-Yekta A. Sternisek P. Weig M. Groß U. Schilling A. F. (2020). Rapid direct detection of pathogens for diagnosis of joint infections by MALDI-TOF MS after liquid enrichment in the BacT/Alert blood culture system. *PLoS One* 15:e0243790. 10.1371/journal.pone.0243790 33306699 PMC7732097

[B29] Otsuka Y. Agestika L. Harada H. Sriwuryandari L. Sintawardani N. Yamauchi T. (2019). Comprehensive assessment of handwashing and faecal contamination among elementary school children in an urban slum of Indonesia. *Trop. Med. Int. Health* 24 954–961. 10.1111/tmi.13279 31192489

[B30] Parvez S. M. Azad R. Pickering A. J. Kwong L. H. Arnold B. F. Rahman M. J. (2019). Microbiological contamination of young children’s hands in rural Bangladesh: Associations with child age and observed hand cleanliness as proxy. *PLoS One* 14:e0222355. 10.1371/journal.pone.0222355 31504064 PMC6736272

[B31] Pasaribu A. P. Alam A. Sembiring K. Pasaribu S. Setiabudi D. (2019). Prevalence and risk factors of soil-transmitted helminthiasis among schoolchildren living in an agricultural area of North Sumatera, Indonesia. *BMC Public Health* 19:1066. 10.1186/s12889-019-7397-6 31391023 PMC6686497

[B32] Pellegrini A. D. (1992). Preference for outdoor play during early adolescence. *J. Adolesc.* 15 241–254. 10.1016/0140-1971(92)90028-4 1447411

[B33] Pessoa R. B. G. de Oliveira W. F. Correia M. T. D. S. Fontes A. Coelho L. C. B. B. (2022). *Aeromonas* and human health disorders: Clinical approaches. *Front. Microbiol.* 13:868890. 10.3389/fmicb.2022.868890 35711774 PMC9195132

[B34] Prüss-Ustün A. Wolf J. Corvalán C. F. Neville T. Bos R. Neira M. (2017). Diseases due to unhealthy environments: An updated estimate of the global burden of disease attributable to environmental determinants of health. *J. Public Health* 39 464–475. 10.1093/pubmed/fdw085 27621336 PMC5939845

[B35] Saeed A. Abd H. Sandstrom G. (2015). Microbial aetiology of acute diarrhoea in children under five years of age in Khartoum, Sudan. *J. Med. Microbiol.* 64 432–437. 10.1099/jmm.0.000043 25713206 PMC4635512

[B36] Satriani S. Ilma I. S. Daniel D. (2022). Trends of water, sanitation, and hygiene (WASH) research in Indonesia: A systematic review. *Int. J. Environ. Res. Public Health* 19:1617. 10.3390/ijerph19031617 35162638 PMC8835571

[B37] Senderovich Y. Ken-Dror S. Vainblat I. Blau D. Izhaki I. Halpern M. (2012). A molecular study on the prevalence and virulence potential of *Aeromonas* spp. recovered from patients suffering from diarrhea in Israel. *PLoS One* 7:e30070. 10.1371/journal.pone.0030070 22355306 PMC3280246

[B38] Shah M. Odoyo E. Wandera E. Kathiiko C. Bundi M. Miringu G. (2017). Burden of rotavirus and enteric bacterial pathogens among children under 5 years of age hospitalized with diarrhea in suburban and rural areas in Kenya. *Jpn. J. Infect. Dis.* 70 442–447. 10.7883/yoken.JJID.2016.398 28250260

[B39] Shakoor S. Zaidi A. K. Hasan R. (2012). Tropical bacterial gastrointestinal infections. *Infect. Dis. Clin. North Am.* 26 437–453. 10.1016/j.idc.2012.02.002 22632648

[B40] Vishwanath R. Selvabai A. Shanmugam P. (2019). Detection of bacterial pathogens in the hands of rural school children across different age groups and emphasizing the importance of hand wash. *J. Prev. Med. Hyg.* 60 E103–E108. 10.15167/2421-4248/jpmh2019.60.2.1186 31312739 PMC6614565

