# Supplementary material for: A comparative pilot study on Gram-negative bacteria contaminating the hands of children living in urban and rural areas of Indonesia versus Germany – A suitable monitoring strategy for diarrhea risk assessment?
Source: Front Microbiol. 2023 Apr 3;14:1152411. doi: 10.3389/fmicb.2023.1152411 (PMC10106674; doi:10.3389/fmicb.2023.1152411)
Supplement: Supplementary file 1 [file Table_1.pdf]

Table S1: Children with contaminated hands and bacterial isolates[1]

Age	Study site	Children tested	Positve children	Bacterial isolates	Enterobacterales	Pseudomonadales	Others
<1	Göttingen	12	2	2	0	1	1
	Medan	20	9	15	7	8	0
	Siberut	14	7	7	6	1	0
1-4	Göttingen	77	35	46	10	32	4
	Medan	80	50	92	41	42	9
	Siberut	77	52	107	45	43	19
5-9	Göttingen	40	20	25	4	17	4
	Medan	40	25	32	10	21	1
	Siberut	40	28	55	23	25	7
10-14	Göttingen	31	8	8	0	7	1
	Medan	40	13	15	3	10	2
	Siberut	40	27	64	23	22	19
All	Göttingen	160	65	81	14	57	10
	Medan	180	97	154	61	81	12
	Siberut	171	114	233	97	91	45

[1]Data indicate absolute numbers of children and bacterial isolates.
